# Skull Vibration-Induced Nystagmus Test (SVINT) in Vestibular Migraine and Menière’s Disease

**DOI:** 10.3390/audiolres11040054

**Published:** 2021-11-08

**Authors:** Roberto Teggi, Omar Gatti, Marco Familiari, Iacopo Cangiano, Mario Bussi

**Affiliations:** ENT Division, San Raffaele Scientific Institute, 20132 Milano, Italy; gatti.omar@hsr.it (O.G.); familiari.marco@hsr.it (M.F.); cangiano.iacopo@hsr.it (I.C.); bussi.mario@hsr.it (M.B.)

**Keywords:** vestibular migraine, Menière’s disease, skull vibration-induced nystagmus test, SVINT, vestibular disorders, episodic vertigo

## Abstract

Background: Vestibular migraine (VM) and Menière’s disease (MD) are the two most frequent episodic vertigo apart from Benign Paroxysmal Positional Vertigo (BPPV) differential diagnosis for them may be troublesome in the early stages. SVINT is a newly proposed vestibular test, which demonstrated to be fast and reliable in diagnoses above all of peripheral vestibular deficits. Methods: We retrieved clinical data from two groups of subjects (200 VM and 605 MD), enrolled between 2010 and 2020. Among others, these subjects were included when performing a SVINT. The purpose of the study is to assess if SVINT can be useful to differentiate the two episodic disorders. Results: 59.2% of MD subjects presented as positive with SVINT while only 6% did so with VM; among other tests, only video HIT demonstrated a different frequency in the two groups (13.1% and 0.5%, respectively), but the low sensitivity in these subjects makes the test unaffordable for diagnostic purposes. Conclusions: Since SVINT demonstrated to be positive in a peripheral vestibular deficit in previous works, we think that our data are consistent with the hypothesis that, in the pathophysiology of VM attacks, the central vestibular pathways are mainly involved.

## 1. Introduction

Vestibular migraine (VM) and Menière’s disease are the two most common causes of episodic vertigo in clinical practice. Diagnoses of both conditions mainly rely on clinical history. Recently, a joint committee of the Barany Society and of the International Headache Society proposed the following criteria for definite VM [1]:

- At least five episodes of vestibular symptoms of moderate or severe intensity, lasting 5 min to 72 h;
- Current or previous history of migraine with or without aura according to the International Classification of Headache Disorders (ICHD);
- One or more migraine features with at least 50% of the vestibular episodes:
  1. Headache with at least two of the following characteristics: one sided location, pulsating quality, moderate or severe pain intensity, and aggravation by routine physical activity;
  2. Photophobia and phonophobia;
  3. Visual aura.
- Not better accounted for by another vestibular or ICHD diagnosis

Furthermore, MD diagnosis mainly relies on clinical history, and audiometrically documented low–medium frequencies sensorineural hearing loss is the main differentiating point [2]:

- Two or more spontaneous episodes of vertigo, each lasting 20 min to 12 h;
- Audiometrically documented low–medium SNHL frequencies in one ear, defining the affected ear on at least one occasion before, during, or after one of the episodes of vertigo with a gap of at least 30 dB at each of two contiguous frequencies below 2000 Hz vs. the contralateral side;
- Fluctuating aural symptoms (hearing, tinnitus, or fullness) in the affected ear;
- Not better accounted for by another vestibular diagnosis.

Migraine is frequently reported in MD subjects, with a frequency ranging from 35% and 56% [3,4]; since cochlear symptoms may be a complaint during vertigo spells also in subjects with VM [5,6], the differential diagnosis between MD and VM in the early stages may be a puzzling dilemma. Electrocochleography (EcoG) and ocular VEMPs have been proposed to be useful to differentiate between the two disorders. Particularly, an increased SP and AP ratio has been more frequently recognized in MD compared to VM subjects (52% vs. 14%) [7], while a reduced ocular VEMPs responses to 500 Hz tone burst compared to 1000 Hz was more frequent in MD subjects [8].

The skull vibration-induced nystagmus test (SVINT) is a newly proposed test which demonstrated to be fast and useful to detect a vestibular asymmetry [9]. In a recent review, it has been reported that, in partial and total unilateral vestibular loss, positivity varied from 60% to 100%, while specificity varied from 62% to 100% [10].

It has been recently published, that in MD subjects, SVINT is positive in around 59% of subjects, with provoked nystagmus most often beating toward the non affected side [11]; moreover, in the follow-up of patients with episodic vertigo without cochlear symptoms, at the onset of disorders, SVINT was predictive for the evolution toward MD [12].

The main purpose of our work was to assess the frequency of a positive SVINT in a wide population of VM subjects, compared to a sample of MD subjects.

## 2. Materials and Methods

### 2.1. Study Cohort

Our sample of VM subjects was composed by 200 adult subjects; data of this retrospective study were obtained from records of patients from 2005 and 2020. The study received ethical approval by an internal committee (No. GO/URC/ER/mm protocol 762). Patients were enrolled if they fulfilled all criteria for definite VM, according to The Barany Society, if all clinical data were retrieved, and if the subjects were followed (and diagnosis confirmed) for at least 3 years. They were excluded if they presented during the follow-up period a sensorineural decrease of hearing level on low to medium frequencies in at least one audiometric exam. Retrospectively, all subjects fulfilled all of Barany Society’s criteria to be included in definite VM.

Clinical data of these subjects were compared with those of a wide sample of 605 definite MD subjects; 322 of these patients were already included in a previous paper [11]. Data were obtained from our records and enrolled between 2010 and 2019. Clinical history was collected with particular attention to migraine. According to International Headache Society (HIS) criteria [13], migrainous headaches typically last between 4 and 72 h and present at least two of the following features: unilateral; pulsating; moderate or severe intensity of pain; and aggravated by, or resulting in the avoidance of, routine physical activity. Cases were excluded if surgically treated before the examination or if they had undergone intratympanic therapy with steroids or gentamicin, and delayed hydrops were not included.

In both groups, bedside examination was performed in a vertigo-free period.

All subjects of both samples underwent central nervous system MRI in order to rule out central vestibular disorders.

Diagnoses of VM-MD, according to the Barany Society’s criteria patients enrolled before 2012–2015, were retrospectively confirmed.

### 2.2. Bedside Vestibular Examination

The presence of nystagmus at various tests was studied with video Frenzel glasses (Interacoustics—Assens—Denmark).

A post-head shaking test was performed with the patients sitting on a chair and the head leaning down of 30°, and nystagmus was observed after 20 shakes of the head with an amplitude around 30°. It was considered positive only when nystagmus lasted for 5 s.

Positional nystagmus was assessed in the supine position with the head turned 90° on both sides.

A skull vibration test (skull vibration-induced nystagmus test—SVINT) was performed at a frequency of 100 Hz with a commercially available system (VVIB—Synapsis). Stimuli were applied perpendicularly to the skin over the mastoid process, with a force around 1 kg while the patient was sitting, and three stimulation trials were performed on each mastoid, lasting 5–10 s each. The test was considered positive only when nystagmus was elicited in all six trials, always beating on the same side. Eye movements were recorded with video Frenzel goggles and visual fixation of both eyes was inhibited.

A video head impulse test (video-HIT) was performed with ICS Impulse, Otometrics, Taastrup, Denmark, only on the horizontal plane. Patients were asked to remove spectacles 5 min before testing and a calibration was performed before tests. Trials with blinks and outliers were excluded. Patients were excluded when eye movements always preceded head movements, even after attempts to improve goggle fit [14]. We considered the exam pathological when VOR gain was lower than 0.80 and/or when corrective saccades were demonstrated, while normal values were detected on the contralateral side.

## 3. Results

### 3.1. Demographic Data

A different sex distribution was demonstrated, since among 200 VM subjects, 174 (87%), while 315 out of 605 (52.1%) in MD sample were females (X^2^ = 78.8; *p* < 0.00001). Furthermore, the age of onset of the first attack of vertigo differed in the two groups: it was 39.2 ± 10.4 in the VM sample and 46.9 ± 14.3 in the MD sample (t = 7.4; *p* ≤ 0.0001).

The duration of attacks in VM sample is summarized in Table 1.

### 3.2. Clinical Data

In the VM sample video, HIT was positive in only 1 subject (0.5%), positional tests in 68 (34.0%), post-head shaking tests in 34 (17.0%), while SVINT in 12 subjects (6.0%). In the MD sample video, HIT was positive in 79 patients (13.1%), positional tests in 225 (37.2%), post-head shaking test in 136 (23.1%), while SVINT was positive in 358 subjects (59.2%). Results and statistics are shown in Table 2. SVINT always presented an horizontal component beating on the unaffected side, while a small torsional component could be seen above all when patients were looking upward.

## 4. Discussion

The differential diagnosis, above all in early stages, between VM and MD may be a puzzling dilemma in some cases. Moreover, migrainous headaches are a common complaint in MD subjects and, on the other side, cochlear symptoms are reported also by VM patients [15]. According to some authors, around 13% of patients meet the diagnosis for both MD and VM [16]. 

In order to differentiate the two disorders, previous authors proposed to perform cVEMPs at 500 and 1000 Hz; a different lower amplitude of responses using 500-Hz stimuli; and possibly partially reduced after glycerol to address the diagnosis toward MD [17,18].

The most significant result in our study is that, among clinical tests, video-HIT and SVINT demonstrated different rates of positivity in the two groups. The first one presented a low sensitivity in both samples, which makes it inappropriate in the diagnostic pathway. On the opposite, SVINT was able to differentiate between the two disorders, since only 6% of VM while 59.2% of MD subjects had a positive test.

SVINT is a practical, simple, and well-tolerated test, and is useful to detect a vestibular asimmetry. It is more sensitive in detecting peripheral vestibular disorders. In total, unilateral vestibular loss was reported to be positive in around 98%, in partial vestibular deficits in 75%, while it resulted positive in only 34% of subjects with central vestibular disorders [9,19]. Recent studies with three dimensional eye recording showed that elicited nystagmus present a torsional and sometimes vertical component, suggesting a global participation of inner ear in observed findings [9,20].

In subjects with vertigo spells without cochlear symptoms, SVINT demonstrated to be useful in predicting evolution toward MD. In another recent paper, a discrepancy between responses to video-HIT and calorics were proposed to be suggestive for MD [12,21].

Different pathophysiology of MD and VM may explain our findings. A peripheral vestibular deficit, provoked by endolymphatic hydrops, is the commonly accepted pathophysiological mechanism of MD, although the relationship between hydrops and the onset of symptoms is far from being clarified [22]. In previous works, video-HIT showed a low sensitivity in detecting vestibular deficits in MD subjects; it can be speculated that video-HIT studies rapid vestibular acceleration, thus focusing on irregular neurons and phasic type-1 hair cells whose activity can be spared in hydrops [23]. On the opposite, SVINT demonstrated to be a more sensitive test in MD subjects, being positive in around 60–70% of MD subjects with a nystagmus beating toward the unaffected side in almost all cases [11,24], although in one previous paper elicited nystagmus in MD subjects could be directed in some cases toward the affected side [25]. In our sample, we elicited a nystagmus always directed toward the unaffected side with a small torsional component (thus with any probability peripheral in origin). It can be hypothesized that the different findings could be related to the fact that our patients were studied in a vertigo-free period.

On the other side, pathophysiology of VM is far from being clarified. Diagnosis mainly relies on clinical history; VM subjects often report a broad spectrum of manifestations, while laboratory findings do not seem consistent with a uniform disorder. Our data support the previous findings of a female preponderance [26]. According to some authors, some of the neurotransmitters involved in pathophysiology of migraine are also expressed in the inner ear and might be involved in VM manifestations [27]. Other authors hypothesized that the reciprocal connections of trigeminal and vestibular nuclei through nucleus raphe magnus, periaqueductal gray, and hypothalamic areas have reciprocal connections which can modulate neural activity within the trigeminal and vestibular system and can cause attacks [28,29]. It is important to note that trigeminal stimulation in migraineurs produces nystagmus, suggesting an increased vestibular excitability in these patients compared with healthy controls [30].

On these bases, it can be hypothesized that the different pathophysiological mechanisms leading to vertigo attacks in VM is the causal factor of a low rate of positivity to SVINT.

Moreover, in our opinion, in controversial cases, it can be useful to differentiate VM from MD [5].

## 5. Conclusions

Data from our study support the hypothesis that SVINT has a high sensitivity to detect a peripheral vestibular deficit in MD outside of a vertigo attack. On the contrary, they are not inconsistent with the hypothesis that a peripheral vestibular disorder can be found in a vertigo-free period in only a few VM subjects.

## Figures and Tables

**Table 1:** Duration of attacks of vertigo in the VM sample. In subjects with different duration of attacks, the most frequent duration was taken.

Duration of Attacks	Number of Subjects
5–20 min	36 (18%)
20 min–4 h	50 (25%)
4–12 h	74 (37%)
More than 12 h	40 (20%)

**Table 2:** Total number and percentage (between brackets) of positivity to various tests across the two samples.

Tests	MD Sample (*n* = 605)	VM Sample (*n* = 200)	*p*
Video head impulse test	79 (13.1%)	1 (0.5%)	<0.00001
Positional tests	225 (37.2%)	68 (34.0%)	0.46
Post-head shaking test	136 (22.5%)	34 (17.0%)	0.12
Skull vibration test	358 (59.2%)	12 (6.0%)	<0.00001

## Data Availability

Since this is a retrospective study, records of these patients ca be found in the system of our institution.
